# Single Nucleotide Polymorphisms in Selected Genes in Inflammatory Bowel Disease

**DOI:** 10.1155/2018/6914346

**Published:** 2018-12-17

**Authors:** Ewa Dudzińska, Magdalena Gryzinska, Janusz Kocki

**Affiliations:** ^1^Chair of Public Health, Medical University of Lublin, 20-093 Lublin, Poland; ^2^Institute of Biological Basis of Animal Production, Subdepartment of General and Molecular Genetics, University of Life Sciences in Lublin, 20-950 Lublin, Poland; ^3^Chair of Medical Genetics, Department of Clinical Genetics, Medical University of Lublin, 20-080 Lublin, Poland

## Abstract

**Introduction:**

Inflammatory bowel disease (IBD) is a complicated, multifunctional disorder characterized by chronic, recurring inflammation of the digestive tract. The two main types of IBD are ulcerative colitis (UC) and Crohn's disease (CD). The aim of the study was to determine single nucleotide polymorphism in fragments of the genes* CARD15/NOD2 *and* DLG5 *in patients from the Lublin Voivodeship.

**Patients and Methods:**

The study was carried out in Lublin (Poland) in 2016. 27 individuals participated in the research. The research group comprised 9 patients with a diagnosis of Crohn's disease and 9 with ulcerative colitis, aged 20 to 48, and 9 healthy volunteers.

**Results:**

No SNPs were confirmed for the* CARD15/NOD2 *gene fragment, but a substitution (T>C) was found in the* DLG5 *gene in a Crohn's disease patient.

**Conclusion:**

Absence of extraintestinal symptoms in patients with Crohn's disease may be associated with the absence of CARD15/NOD2 SNPs. The study suggests that SNPs (T>C substitution) affect the function of the DLG5 protein and thus play a role in the development of IBD, in particular Crohn's disease. The analysis presented is a pilot study due to the small number of samples.

## 1. Introduction

Inflammatory bowel disease (IBDs) is a complex, multifunctional disorder characterized by chronic, recurrent inflammation of the digestive tract [1]. The two main types of IBD are ulcerative colitis (UC) and Crohn's disease (CD). These two diseases are a major health problem throughout the world. Their prevalence in Europe is 12.7 and 24.3 per 100,000 individuals, respectively, and is steadily rising among children and adults around the world [2].

Extensive research conducted over the last few decades suggests that genetic predisposition and environmental factors can lead to malfunctions of the epithelial barrier and consequent deregulation of the mucosal immune system and an abnormal response to intestinal microbiota [3]. These are therefore varied interactions between genetic, bacterial, and environmental factors. The latest research on genetics and immunology has confirmed that the innate immune system is of great importance in inducing intestinal inflammation [2]. The* CARD15/NOD2* gene (nucleotide-binding oligomerization domain-containing protein 2/caspase recruitment domain family number 15) is a gene that provides a defensive strategy through the innate immune system to protect hosts against bacterial infection [4].* CARD15/NOD2* plays an important role in immune function. In response to bacterial infection,* CARD15/NOD2* acts as an intracellular bacterial receptor and activates the kappa B nuclear factor (NF-*κ*B), particularly after recognizing the bacterial wall component muramyl dipeptide (MDP) [5]. One of the cell types in which high expression of the*CARD15/NOD2* gene has been observed is Paneth cells, most of which are located in the terminal ileum. Paneth cells play an important role in the innate regulation of the intestinal microbiota by synthesizing and secreting peptides or antimicrobial proteins such as lysozyme or *α*-defensins.* NOD2/CARD15* mutations lead to dysregulation of host–microbe interactions, contributing to the development of inflammation in the ileum, which is characteristic of Crohn's disease [4]. The gene*DLG5* (discs large homologue 5), on the other hand, encodes scaffold proteins belonging to the MAGUK family, which participate in the formation of cellular connections, maintenance of cell shape, and intracellular signal transduction [6]. Expression of this gene is widely expressed in the tissues of the small and large intestines.* DLG5* gene polymorphisms have been shown to increase susceptibility to IBD, including both CD and UC. [7].* DLG5* has been shown to be localized at cell–cell contact sites and is involved in maintaining epithelial integrity. Different variants of* DLG5* may contribute to the loss of cell polarization complexes and adhesion complexes, so that epithelial cell polarity is not maintained and epithelial-mesenchymal transition (EMT) is induced [7]. EMT is a process involving the transformation of immobile, polarized cells with an epithelial phenotype into cells with a mesenchymal phenotype. The characteristic features of EMT include lack of polarity and cell adhesion, reduced expression of E-cadherin, and increased mobility and invasion capacity [8]. Thus, it can be assumed that* DLG5* polymorphisms may impair the epithelial barrier in the gastrointestinal tract and lead to abnormal epithelial structure, making it more susceptible to IBD (CD and UC). Furthermore, research by Friedrichs et al. has shown that the DLG5 scaffold protein also belongs to the CARD family of proteins (likeCARD15/NOD2). Thus, DLG5 is probably involved in the regulation of NF*κ*B activation or caspase activation within the host defence mechanisms. Therefore, both the* NOD2 *and* DLG5* genes may interact functionally to contribute to the risk of developing CD [9].

This paper attempts to show polymorphisms of the* CARD15/NOD2* gene and the* DLG5* gene in patients with IBD, including CD and UC, which may have contributed to the development of the disease in patients from the Lublin Voivodeship.

## 2. Materials and Methods

The research was carried out in 2016 at the Cardinal Stefan Wyszynski Regional Hospital in Lublin. 27 individuals participated in the research. The study group consisted of 9 patients diagnosed with Crohn's disease and 9 with ulcerative colitis, aged 20 to 48 years. The test material was blood collected from patients on an empty stomach after 12 hours of rest. In addition, medical history was taken with regard to the occurrence of extraintestinal symptoms and autoimmune diseases in the family. The family history of all subjects was negative. The control group consisted of 9 healthy individuals.

DNA was isolated using a QIAamp DNA Blood Mini Kit (QIAgen), followed by quantitative and qualitative evaluation of the isolated DNA samples. The measurements were performed by UV-Vis spectroscopy using a Biophotometer (Eppendorf). Electrophoresis was carried out in a 1% agarose gel with a constant current of 60V applied for 1 hour.

The following primers were used for PCR: (F) GACTCTTTTGGCCTTTTCAGATT and (R) CCAATGGTCTTTTTTCCTTACTCC for* CARD15/NOD2* and (F) TTATTCCCCTTCCACAGGCACTAC and (R) GCCGCAGCTGAATGGAGA for* DLG5* [10]. The reactions (20 *μ*L total volume) contained 5*μ*l DNA (with a DNA concentration of 50 ng/*μ*l) and 1.0 U Taq polymerase (AmpliTaq Gold 360 DNA Polymerase, Applied Biosystems) in the manufacturer's buffer, adjusted to a final concentration of 2.5 mM MgCl_2_, 0.2 mM of each dNTP, and 0.1 mM of each primer. PCR cycling conditions were 95°C for 7 min; 30 cycles of 95°C for 60 s, 55°C for 60 s (*CARD15/NOD2*) or 59°C for 60 s (*DLG5*), 72°C for 60 s; and 72°C for 10 min (Labcycler, SensoQuest).

The PCR product was sequenced and the sequences obtained were recorded in FASTA format. The nucleotide sequences of the* CARD15/NOD2* and* DLG5* gene fragments were compared using DNA Baser software.

The approval of the Bioethics Committee at the Medical University in Lublin was obtained for the study (approval no. KE-0254/179/2016).

## 3. Results

### 3.1. Spectrophotometric Evaluation

The DNA concentration in the test samples was high, ranging from 18 to 41 ng/*μ*l. The purity of the samples (mainly protein contamination), defined as the 260/280 nm absorbance ratio, was satisfactory.

### 3.2. Electrophoresis of PCR Products

The results of electrophoresis of PCR products from the patients indicate that the size of the amplified fragment of the* CARD15/NOD2* gene was 243 bp, while that of* DLG5 *was 107 bp. The samples were sequenced.

### 3.3. Genetic Analysis

#### 3.3.1. CARD15/NOD2

**Figure 1:**
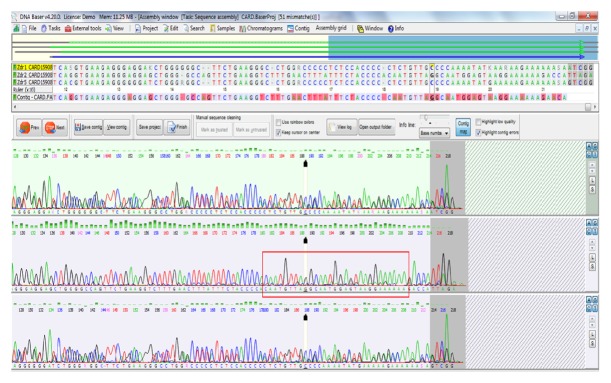
Chromatographic record for the* CARD15/NOD2* gene fragment for a healthy subject (from the control group).

The size of the analysed* CARD15/NOD2 *gene fragment was 243 bp. No SNPs were observed in this fragment in patients with CD or UC. The gene fragment in the control group was interesting. Over a length of 28 nt (from 180 to 208 of the sequenced fragment), the sequence was entirely different from the sequence in the database [Figure 1]; however, this sequence was homologous with the complementary strand, which may be evidence of translocation. This may indicate DNA inversion in this fragment, especially since complete nucleotide homology with the complementary strand (Crick strand) was confirmed.

#### 3.3.2. DLG5

**Figure 2:**
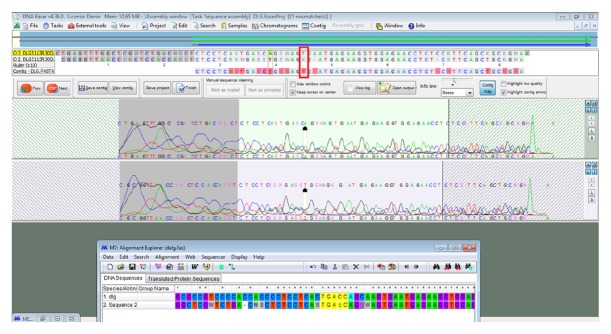
SNP at position rs1248696, T>C substitution in a patient with Crohn's disease.

The size of the* DLG5* gene fragment was 107 bp. One SNP at position 1248696 was found in the gene fragment. A T>C substitution occurred in one sample (patient 3) from the group of patients with Crohn's disease [Figure 2].

## 4. Discussion

Intensive research conducted in the search for genetic factors underlying the etiopathogenesis of inflammatory bowel disease has resulted in the identification of the* CARD15/NOD2* gene, which is associated with increased predisposition to Crohn's disease. Four polymorphisms of the* CARD15/NOD2 *gene that increase predisposition to developing CD (up to 40 times) have been identified: G908R, R702W, 3020insC, and 802C/T, the last of which is frequently observed in Poland [11, 12].

In the fragment of* CARD15/NOD2* analysed in the present study, no SNP position was found in patients with CD or UC. However, Strober et al. emphasize that polymorphisms in the* CARD15/NOD2* gene are significantly associated with dysregulation of the mucosal immune response to commensal microorganisms.* CARD15/NOD2* is a member of the NOD-like receptor family (NLR) and is a receptor for bacterial peptidoglycan. Thus* CARD15/NOD2* polymorphism is associated with an inappropriate immune response to intestinal bacteria, which in turn leads to quantitative or qualitative changes in the bacterial population in the intestinal lumen or lamina propria, causing inflammation.* CARD15/NOD2* mutations have been shown to lead to decreased intestinal production of *α*-defensins by Paneth cells and to a loss of immune response to pathogens.* CARD15/NOD2 *dysfunction has also been shown to lead to the development of CD by inducing changes in the intestinal microflora, thereby affecting immune effectors. There are also reports that* CARD15/NOD2 *dysfunction may cause immunoregulatory dysfunction of the innate immune response, which may also lead to the development of inflammatory bowel disease [13].

As pointed out by Zatorski et al., polymorphisms of the* CARD15/NOD2* gene encoding the LRR region are one of the important genetic factors predisposing to CD, with three of them, Arg702Trp, Gly908Arg, and Leu1007insC, accounting for approximately 82% of mutant alleles. The authors note that a study conducted on a mouse model lacking the CARD15/NOD2 protein found greater susceptibility to* Listeria monocytogenes* infection. This confirms that CARD15/NOD2 protein dysfunctions cause both quantitative and qualitative changes in the microflora of the terminal ileum, which leads to the development of an inflammatory process similar to CD, induced by the entry of bacteria into the lamina propria [14].

Although numerous reports confirm that* CARD15/NOD2* gene polymorphisms are associated with a predisposition to IBD, our research did not show this relationship. This may be linked to the absence of parenteral symptoms in the subjects. Szeliga et al. stress that three polymorphisms of the* CARD15/NOD2* gene are particularly associated with the risk of CD. These are SNP8 (R702W), located between the NOD domain and the first LRR sequence, and SNP12 (G908R) and SNP13 (1007fs), which are located within the sixth and tenth LRR sequences. The occurrence of mutations in both gene alleles (homozygosity) or the occurrence of different mutations within one or both alleles (complex heterozygosity) increases the risk of CD about 40-fold. A single mutation in one allele (heterozygosity) increases the risk 2 to 4 times. One of the most common single nucleotide polymorphisms of the* CARD15/NOD2* gene is P268S (SNP5), where the cytosine residue at position 802 is replaced by thymine. In the Polish population, P268S polymorphism has been found in 49.5% of patients with CD, and its presence in both alleles is associated with earlier onset of disease symptoms and increased risk of parenteral symptoms such as joint abnormalities, iritis, and erythema nodosum. P268S polymorphism has also been demonstrated in patients with the 1007fs mutation (14.9%) [15]. The patients in the present study did not report any parenteral symptoms, which may be linked to the lack of polymorphisms in the analysed gene fragments.

The other gene analysed in our study was* DLG5*, which plays an important role in maintaining epithelial structure. Genetic variants of* DLG5* affect the function of the intestinal epithelial barrier.* DLG5* contains one DUF622 domain, four PDZ domains, and one SH3 domain, followed by the guanylate kinase-like domain. All of these domains are believed to be involved in protein–protein interactions [16]. The authors cited conducted an* in silico* analysis of the potential structural and functional implications of the R30Q and P1371Q variants. The results suggested that both variants are likely to impair the function of DLG5 scaffolds. This is also supported by our research, which found an SNP polymorphism at position 1248696. A T>C substitution was found in the analysed fragment of the* DLG5* gene in the group of CD patients. Other authors note that the R30Q (Rs1248696) variant of* DLG5*, where amino acid 30 in exon 3 changes from arginine to glutamine, is associated with the development of IBD [17]. Reports by Sezaki et al. indicate that polymorphisms in* DGL5* are associated with CD in a sex- and age-dependent manner [18].

Lin et al. confirmed that DLG5 P1371Q was associated with IBD in both sporadic and familial IBD patients from the population of central Pennsylvania. The authors suggest that SNPs affect the function of the DLG5 protein and thus play a role in the development of IBD. Two synonymous SNPs, R30Q and P1371Q, were shown to be significantly associated with IBD, whereas no such association was found for the* DLG5*SNP G1066G. The study included both the familial and sporadic IBD population from central Pennsylvania (USA), in which a genetic link between R30Q and IBD was demonstrated. Our study also demonstrates a relationship between* DLG5* R30Q (rs1248696) polymorphism and the development of IBD [17].

Lin et al. note that there is increasing evidence that epistasis can play an important role in the emergence and development of human diseases. In the case of epistasis, the presence of two or more specific loci may increase or decrease the risk of disease more than would be expected from their independent effects [17].

The* DLG5* gene contains many functional domains distributed throughout the gene, such as leucine zipper and the PDZ1, PDZ2, SH3 and GUK domains [17]. Other researchers have shown that in* DLG5 *the nonsynonymous single nucleotide polymorphism 113G → A results in the amino acid substitution R30Q in the DUF622 domain of DLG5. This mutation probably impairs scaffolding, signal transduction, and intestinal epithelial cell integrity. In addition, the additional presence of polymorphisms of* CARD15*, i.e., Arg702Trp, Gly908Arg, and 1007fs, has been shown to significantly increase the risk of IBD, which suggests a potential interaction between the two genes. The gene–gene interaction between* DLG5* and* CARD15/NOD2* reflects the complex nature of polygenic diseases [16].

Our analysis did not confirm a relationship between the* CARD15/NOD2* and* DLG5* genes, as polymorphism was noted in only one gene,* DLG5*. This may be due to the small size of the study group. Therefore further research is needed to show potential interactions between the* CARD15/NOD2* and* DLG5* genes.

## 5. Conclusions

The analysed variants of the* CARD15/NOD2 *and* DLG5* genes can significantly contribute to the development of IBD, i.e., Crohn's disease and ulcerative colitis. Absence of extraintestinal symptoms in patients with Crohn's disease may be associated with the absence of CARD15/NOD2 SNPs. The study suggests that SNPs (T>C substitution) affect the function of the DLG5 protein and thus play a role in the development of IBD, in particular Crohn's disease. The analysis presented is a pilot study due to the small number of samples.

## Data Availability

The data used to support the findings of this study are included within the article.
